# The implications of decolonization on China’s academic global health: a dialogue with Stephen Gloyd at the Luhu Global Health Salon

**DOI:** 10.1186/s41256-023-00299-x

**Published:** 2023-05-17

**Authors:** Yu Tang, Feifei Zhang, Dong Roman Xu

**Affiliations:** 1 Acacia Lab for Implementation Science, School of Health Management and Dermatology Hospital, Southern Medical University, Guangzhou, China; 2 Center for World Health Organization Studies and Department of Health Management, School of Health Management of Southern Medical University, Guangzhou, China; 3 Southern Medical University Institute for Global Health (SIGHT), Dermatology Hospital of Southern Medical University (SMU), Guangzhou, China

**Keywords:** Academic global health, Decolonization, Equity, Chinese university

## Abstract

The call for decolonization in global health is growing alongside China’s increasing involvement in the field. This perspective paper presents and extends with a further literature review of a dialogue with Stephen Gloyd, a global health professor from the University of Washington, conducted in July 2022 at the Luhu Global Health Salon. Drawing from Gloyd’s four decades of experiences in low- and middle-income countries, as well as his role in creating the University of Washington’s global health department, the doctoral program in implementation science, and the non-governmental organization, Health Alliance International, this paper delves into the concept of decolonization in global health and explores how Chinese universities can expand their participation in global health while striving for equity and justice. Focusing on China’s academic global health research, education, and practice, the paper proposes specific recommendations for building an equity-focused global health curriculum, addressing power imbalances and inequalities in university-affiliated organizations, and strengthening South-South cooperation in practice. The paper offers implications for Chinese universities on expanding future global health cooperation, promoting global health governance, and avoiding recolonization.

## Background

In recent years, China has taken on a more proactive role in global governance by proposing initiatives such as “a Community of Shared Future for Mankind” [1], “the Belt and Road Initiative” [2], and “A Global Community of Health for All” [3]. As part of this shift, China has integrated itself into the world health system as an aid recipient and an aid provider while taking on more international responsibilities [2]. China has contributed the second-highest amount of membership fees to the United Nations and has committed to supporting multilateralism centered on global governance [4]. However, China is in shortage of global health professionals to engage in global health [5, 6]. In this context, the Luhu Global Health Salon was launched as a collaborative initiative between the Southern Medical University Institute for Global Health (SIGHT) and the Chinese Consortium of Universities for Global Health (CCUGH). This platform invites distinguished global health experts to share their experiences and insights on various topics related to global health through engaging dialogue sessions. The Salon collaborates with the Journal of Global Health Research and Policy (GHRP) to produce perspectives based on each Salon dialogue.

This article, the first in the series, summarizes critical points of the dialogue with Professor Steve Gloyd, MD, MPH, of the University of Washington (UW) on July 23, 2022. The article also expands the discussion with a literature review. Professor Gloyd is a family physician and has been a UW faculty member since 1985. With over 40 years of experience, he has worked as a clinician, manager, researcher, teacher, and policy advocate, with a focus on improving Primary Health Care, maternal-child health, tuberculosis and malaria control, and STD/AIDS in various parts of the world. Through this dialogue, Gloyd described his academic career and shared his practical experiences in global health in Mozambique, Côte d’Ivoire, and many other places. He also described the development of UW’s global health and implementation science programs and his founding of the Health Alliance International (HAI). The dialogue focused on the issue of decolonization in global health partnerships and potential lessons learned for China’s academic community working in global health. China’s academic global health referenced in this paper mainly refers to research, teaching and practice in global health conducted by Chinese universities or other research entities.

## Decolonizing global health: addressing power imbalances and colonialism

Gloyd asserted that decolonizing global health requires acknowledging the presence of colonialism and power imbalances. He noted that characteristics of colonialism still exist in global health, particularly in high-income countries (HICs), perpetuating control and domination over low- and middle-income countries (LMICs) in a manner that echoes the colonial era.

Global health is an interdisciplinary field that emerged from medicine, public health, and international health, focusing on health equity and mutual benefits [1]. The roots of global health can be traced back to tropical medicine during colonial times, which focused on studying the infectious diseases prevalent in colonial tropical territories to protect colonists from these diseases and to help ensure an adequate workforce for colonial extraction [7–9]. With the independence of colonies in Asia, Latin America, and Africa in the mid-20th century [10], tropical medicine evolved into international health and, eventually, global health [9]. However, as Gloyd argued, major aspects of colonialism are still evident, and global health can sometimes be just a new label for old practices [11]. The persistent power imbalances, socio-economic inequities, racism, and new colonialism contribute to global health inequities [12]. HICs in the North are often viewed as centers for top-quality global health education, training, and research. They are home to leading global health journals, influential International Civil Society Organizations, and private foundations. As a result, individuals and institutions in HICs have greater access to educational and research funding opportunities, better chances for publication, and more resources than their counterparts in LMICs [11]. Most health aid projects are still donor-driven rather than guided by the priorities of researchers and communities in recipient countries [13]. English remains the dominant language in global health research [14]. Exacerbating the problem, researchers from LMICs often seek better resources by leaving to work in HICs, leading to a loss of talent and further weakening the research capabilities, workforce, productivity, and sustainability of LMICs [15].

According to Gloyd, decolonizing global health requires a collaborative effort from both HICs and LMICs and entails funding, communication, and ideology changes. Global North donors must acknowledge the power imbalance stemming from their economic and technological advantages, while Global South recipients must recognize that they can reverse the power imbalance and that, most of the time, they do not need to be reliant on external support.

Recent literature and discussions have highlighted global health inequity between the North and South. The challenges of decolonization require a thorough reevaluation of long-standing practices, including funding structures, unequal systems, and even mindsets [11–13, 16]. Yerramilli and colleagues agree that LMICs are often not lacking in funds and capability to address their health issues. There is an outflow of funds from LMICs to HICs that exceeds the medical aid provided by HICs, and more qualified health personnel are flowing to HICs, becoming the central workforce in their healthcare systems [17]. Regardless, unequal economic and research capacities between LMICs and HICs further exacerbate the dependency psychology of LMIC researchers and the ‘savior’ mentality of HIC researchers, reinforcing the reliance on the Global North [9, 18]. Additionally, colonial thinking remains prevalent, making us less sensitive to remnants of colonialism in daily practices and organizational structures [9, 11]. Therefore, decolonizing global health requires a continuous and collaborative struggle between the Global North and South to change long-standing funding structures, unequal systems, and colonial mindsets.

## China’s growing role in global health

Gloyd commented that China’s increasing involvement in global health could be attributed to its status as the world’s second-largest economy and leading manufacturing country, with a significant presence in Africa and Asia. China has experience in strengthening its health systems in impoverished areas and coordinating medical resource allocation, making it a valuable partner for aid and technology exchange.

China’s global health engagement is dominated by government activities. In contrast to the path of the Global North which has a historical linkage to colonization, China’s foreign aid is based more on consolidating political friendship and promoting mutual economic and social benefits [19, 20]. China provided foreign aid totaling RMB 270.2 billion (39.3 billion USD) from 2013 to 2018, with Africa receiving 44.7%, Asia receiving 36.8%, and Latin America receiving 7.3% [21]. China’s aid in health encompasses five categories: medical teams, construction of health facilities, donation of drugs and equipment, training of health personnel, and infectious disease control [19]. From 1963 to 2014, the Chinese government sent about 23,000 Chinese medical workers to around 66 countries, serving approximately 270 million people [2, 19]. Over 140 medical infrastructure projects were constructed from 2010 to 2018, primarily as donations [19, 21]. In particular, China has accelerated the construction of the Africa Centers for Disease Control and Prevention (Africa CDC) and dispatched disease control experts to strengthen African public health systems [21]. In response to the Ebola outbreak in West Africa, China provided emergency humanitarian assistance of $120 million to 13 African countries and sent nearly 1,200 medical and public health personnel to epidemic-affected countries, training 13,000 medical personnel [2, 21]. China provided technical assistance to Tanzania to control schistosomiasis, reduce the risk of infection, and implemented a fast-acting artemisinin project in Comoros, reducing the number of malaria cases by 98% [21].

Recently, Chinese universities have been playing an increasingly important role in China’s global health activities [19]. Many universities have established centers or programs in global health. In November 2013, ten universities, including Central South University, Duke Kunshan University, Fudan University, Kunming Medical University, Peking University, Peking Union Medical College, Sun Yat-Sen University, the Chinese University of Hong Kong, Wuhan University, and Zhejiang University, established the Chinese Consortium of Universities for Global Health (CCUGH) [22]. These universities have been critical in providing China’s educational and training opportunities for government officials, technical personnel, and students from developing countries. Examples include the Chinese Government Scholarships, the Chinese Trade Unions Silk Road Scholarship, MORCOM SCHOLARSHIP-CSC Program, and the ASEAN-China Young Leaders Scholarship. In 2018, China received 492,185 international students, among whom 12.0% were medical students and 63,041 (12.8%) were awarded the Chinese government scholarship [23, 24]. Chinese universities are also the main driving force behind global health research. China has accumulated rich health development experiences, such as the barefoot doctor model and the three-tier rural health system in the 1950s, which inspired the primary care movement in Alma Ata [19, 25]. More recently, China’s rapid reconstruction of rural health insurance and “health for wealth” poverty alleviation initiative have provided valuable lessons for the Global South. Universities play a vital role in summarizing and transforming these Chinese experiences in health system development.

## Implications of decolonization for Chinese academic global health

As China engages more in global health, Gloyd stressed the importance of embracing decolonization by the Chinese to be responsible and effective players in the field. With China’s position as a major player in South-South relationships, it can approach global health issues from a decolonization perspective. As both a recipient and donor country, China has an opportunity and responsibility not to replicate unequal power dynamics in the historical Global North-South partnerships.

However, decolonization has thus far received limited attention in China. Some may be complacent, assuming that since China has never colonized any country, decolonization does not apply to them. Nonetheless, Kwete and other Chinese scholars have made efforts to raise awareness of decolonization issues and provide guidance for related problem-solving [11]. While Chinese universities may not have direct colonial experience, it is essential for them to recognize the remnants of colonialism and be vigilant of the potential for colonial consciousness in future global health activities. Moreover, as citizens of a historically semi-colonized and formerly aid recipient country, Chinese scholars have a responsibility to actively promote the global health decolonization movement, establish a new model of South-South cooperation, and contribute to the formation of global health solidarity based on the equity of all stakeholders. The Chinese government’s commitment to promoting equitable and balanced partnerships in global development provides valuable guidance for Chinese universities participating in global health decolonization in the early stages [21].

### Decolonizing global health education: equity-focused curriculum

In 1987, Gloyd launched the International Health MPH Program at the UW. He played a key role in the creation of the UW Department of Global Health in 2007 which is situated in both the UW Schools of Medicine and Public Health. Gloyd noted that during the early stages, there were two competing views on the priorities for the department: one that emphasized bio-medical research and the other that focused on both research and practice in Primary Health Care, social justice, community health, and implementation science. While bio-medical and epidemiologic research remains the prominent funding source, the academic structure of the department ensures that UW courses address health systems, the social, economic, and political drivers of global health, and the values of justice and equity. The curriculum is designed to build a social equity framework for cultivating an effective cadre of global health workers from the Global North and Global South.

The 1990s saw a proliferation of academic discussions on global health aimed at more effectively addressing global health issues [1]. Many universities in the US, UK, and Canada established international or global health programs [26, 27], leading to an increased focus on training global health professionals from around the world. Since then, many scholars have reflected on how to achieve equity and decolonization in global health education. Eichbaum et al. proposed specific measures to decolonize global health education, following the Association of American Colleges and Universities (AAC&U) Global Learning VALUE and the Consortium of Universities for Global Health (CUGH) Competencies Toolkit, emphasizing equity in education program implementation and educational opportunities and increasing intercultural communication and practice [8]. Jacobsen et al. suggested a “5 Ps model of global health education”, which outlines a comprehensive framework encompassing parity, people, planet, priorities, and practices [28].

Over the past decade, Chinese universities have begun to offer global health education and research. Peking University established China’s first department of global health at its School of Public Health in 2012. Wuhan University began offering an undergraduate program in global health in 2012 and a master’s degree in 2014 [29]. In 2014, Wuhan University and Duke University jointly established Duke Kunshan University, which offers a Master’s program in global health. With the support of China’s Ministry of Commerce, Southern Medical University began offering its international MPH program in 2015, admitting approximately 25–40 students each year from LMICs with full scholarships. Other universities in China, such as Fudan University, Sun Yat-sen University, Central South University, and Peking Union Medical College, have also established programs in global health [19].

Global health education in China has mainly been led and developed by the public health sector, taking a less biomedical approach. However, the current curriculum of undergraduate and graduate programs does not sufficiently emphasize equity, nor is it tailored to meet the challenges faced by LMICs. Wuhan University has developed a program for global health professionals that considers the conditions in China, consisting of four core modules: clinical medicine, preventive medicine, global health, and related courses [29]. The program integrates medical knowledge and humanistic values and emphasizes a multidisciplinary approach to addressing global health issues. However, it falls short in terms of equity compared to US universities such as Kent State, Georgetown, and the UW, all of which emphasize improving the health of disadvantaged populations and require students to identify and analyze health problems in low-service areas and underserved populations [30, 31].

Addressing health inequities is a fundamental ethical principle in global health; thus, Chinese global health education would do well to create a culture that explores how to advance health equity from a national and international perspective. Equipping students with the ability to identify and solve important health problems among different populations is also critical, particularly in diverse low-income and resource-poor areas [30]. Reflecting on the UW experience presented by Gloyd and drawing on the tools proposed by Eichbaum and Jacobsen et al., Chinese universities can strengthen their programs by critically evaluating existing education programs and assessing the degree to which the colonial mentality has infiltrated their teaching and research. Doing so can promote a more equitable power dynamic with students and collaborators in the Chinese global health academic environment.

### Decolonizing global health funding: addressing power imbalances and prioritizing equity in university-affiliated organizations

Gloyd highlighted the significant diversion of global health funding, with 60–80% of aid supposedly allocated to LMICs ending up supporting the technical and administrative staff and expenses of big international Non-government Organizations (NGOs) and universities. Moreover, several studies have documented the mechanisms of power and funding inequities among collaborating research institutions in HICs and LMICs. As an illustration, the US National Institute of Health Fogarty International Center awarded 70% of its grants to the US and other HICs, while 73% of Wellcome Trust grants supported activities in the UK. An estimated 88% of Bill and Melinda Gates Foundation grants also went to institutions in the Global North [18]. This funding and power imbalance means that HIC researchers and institutions have the authority to decide how funds are used, set local priorities, control data and knowledge ownership, and publish research results, leading to parachute research. In contrast, most of the funds are used to pay HIC researchers’ salaries with little compensation for LMIC researchers [18, 32, 33]. In 2018, countries in the WHO African region received less than 1% of global research funds, further hindering their research capacity and exacerbating power dynamics [34].

Drawing on Gloyd's Health Alliance International (HAI) experience, where donors disproportionately support large NGOs compared to government ministries of health, senior public sector health officials frequently leave their posts for the better-funded NGOs. Thus, ministries of health are weakened both by inadequate funds to cover recurrent costs and the internal brain drain of critical human resources to the NGOs. Gloyd proposed that university-affiliated organizations should implement agile and adaptable financing and organizational methods. This could be achieved by collaborating with aid-receiving countries or smaller NGOs and reducing the reliance on these organizations for technical support of faculty, staff, and students. Gloyd believes Chinese universities have a unique advantage in this regard since faculty salaries are paid mainly by the university, thus reducing the corresponding personnel and administrative costs. To learn the lessons of the imbalance in HIC funding mechanisms and to ensure that most resources are directed towards the aid recipient, Gloyd recommended that Chinese university-affiliated organizations establish transparent funding and expenditure plans, with a focus on preventing donors and their NGOs from absorbing excessive resources that should be directed to the government of the aid-receiving country.

There is an increasing call for equitable funding in global health, including several specific actions for improvement [34–36]. Charani et al. suggested that donors design more optimized donation systems, track the benefits of aid recipients, distribute all resources fairly and fully, optimize funding structures, empower those who are affected unequally, and strengthen reflection and feedback. They proposed a framework for self-assessment by donors, covering the entire process of planning, implementing, monitoring, and evaluating aid. This framework can be used to assess ownership, partnerships, and fairness in the aid process [18]. Kumar et al. emphasized the need for increased transparency in funding for global health research and for researchers in recipient countries to take a leadership role in controlling funds and resources [16]. Hodson et al. suggested prioritizing direct funding for researchers in LMICs and establishing mechanisms for LMIC institutions and researchers to lead direct funding [12]. These recommendations not only support Gloyd’s observations but also offer practical guidance for future Chinese endeavors. Chinese universities and their affiliates have so far limited engagement in global health, with most activities taking place within China, such as providing global health education and policy advice [6, 37]. However, they have been experimenting and becoming increasingly active in direct operations, such as research fieldwork in partner countries. Due to their relative lack of experience, it is critical for Chinese universities to learn from the rich history of Global North-South global health work and prevent the emergence of an inequitable power dynamic in future practice [18].

Gloyd also stressed the importance for university affiliates to recognize their role as partners with recipient countries, respecting local needs, and working on an equal basis to avoid any resemblance of recolonization in the relationship between the organization and the recipient. Nakanjako et al. also highlighted that partnerships are formed because no organization or group can achieve its objectives in isolation. Establishing effective and sustainable partnerships requires all partners to be committed to equality from the outset and fully aware of the challenges that may undermine good relationships [36]. Steenhoff et al. proposed that mutually beneficial relationships lie at the core of successful partnerships [38]. These perspectives align with the Chinese government’s push for establishing a more equitable and balanced global development partnership, which should also become an important criterion for Chinese university global health affiliates in pursuing future partners.

### Decolonizing academic global health practice: enhancing South–South collaborations

Gloyd described the highly competitive nature of the global health field, where big NGOs and universities frequently fight with each other for large sums of aid and research money. Many workers and their global health institutions greatly benefit from substantial aid funding, and they tend to protect their turf. Institutional conflicts and power dynamics are challenging to change, whether they are donors, universities, or NGOs. Nevertheless, he emphasized that the Chinese academic community has ample opportunities to pursue more equitable South–South partnerships that might contribute to a more collaborative and compassionate environment among partners. He suggested that one of the keys is to enable more Chinese individuals to work on the ground in recipient countries to gain knowledge of the subtleties of life and earn the trust of the LMIC collaborators to craft genuine partnerships.

Despite China’s increasing involvement in global health, its overall impact is still relatively new and untested [6, 19]. Lack of practical experience in LMICs and inexperienced aid management systems often limit the effectiveness of Chinese institutions [6, 37, 39]. From 2012 to 2019, the China-UK Global Health Support Programme (GHSP), funded by the UK Department for International Development (DFID) to the National Health Commission in China, provided crucial experience for the Chinese government and research institutions to diversify their participation in global health and establish participation mechanisms [39]. The program, however, had limited participation from universities [6]. More recently, with the support from the Swiss Agency for Development and Cooperation (SDC), Southern Medical University, along with other domestic and foreign partners, such as Lanzhou University, Sichuan University, Central South University, Guizhou Medical University, Inner Mongolia Medical University, Kathmandu University, the Mozambique Health Committee, and the Ministries of Health of Nepal and Mozambique, jointly launched the Silk Road Labs for Health Systems Strengthening (S-Labs). This program aimed to strengthen health systems by applying implementation science methods in those countries along “the Belt and Road”.

Gloyd suggested that Chinese universities, jointly with their LMIC partners, should establish clear and transparent support mechanisms to promote the implementation and evaluation of global health projects while avoiding HICs’ “parachute research” mentioned above. Hodson et al. highlighted the importance of pairing Principal Investigators (PIs) from HICs and LMICs as co-PIs to provide local solutions to local issues. This approach enables LMIC institutions to take ownership of and manage the activities [12]. Abouzeid et al. suggested a sustainable system to strengthen global health leadership in the Global South at various levels, including researcher, institutional, and organizational. This system promotes mutual benefit, capacity exchange, and the development of new partnership models for co-leadership. They also advocated for promoting South–South cooperation, particularly between promising institutions in stronger Global South countries and weaker institutions in less developed countries [40]. These scholars offer valuable systematic and macro-level suggestions. In terms of strengthening South–South cooperation and leadership in the Global South, Chinese universities have made some attempts by training LMIC government officials and technical staff under the guidance of the Chinese government. However, due to the lack of practical experience and partners/networks in LMICs [6], the above recommendations still have important implications for Chinese academic global health practice.

Gloyd emphasized the importance of real-world implementation science as an excellent way to engage in research and enhance collaboration. He pointed to the S-Labs program as an example of how health departments or academic institutions can be supported in a humble and open-minded manner. Implementation science is an interdisciplinary field that is rapidly growing. Its main goal is to support the adoption, implementation, and sustaining of evidence-based best practices, particularly in LMICs, where health inequities and implementation barriers are significant [41, 42]. Shelton et al. argued that since LMICs are home to 80% of the global population, they face more significant health inequities and implementation barriers, making them promising locations for implementing research. The practical application of implementation research requires researchers to establish long-term and trusting partnerships with local community stakeholders and patients to improve local health and health care [41]. Indeed, for Chinese research universities, using implementation science is an excellent starting point to promote China’s experience of Primary Health Care and enable closer and more positive relationships with the recipient community more humanely and equitably. It is also an effective pathway for South-South cooperation.

Gloyd noted the potential and valuable contribution of China’s increasing number of international students from Asia, Africa, and Latin America to global health research and practice in China. By selecting exceptional Chinese-speaking students, universities can establish a local talent pool that can play an essential role in global health initiatives built on the trust and solidarity fostered through educational relationships. This approach of actively developing deep friendships and collaboration with individuals in recipient countries is recommended by Gloyd based on his experience in LMICs such as Mozambique, Kenya, Peru, and Sudan. Moreover, improving the equity of global health partnerships and developing personal connections among team members can effectively create, solidify, and sustain long-term partnerships [43, 44]. Chinese universities have already implemented this approach in their health practices, such as in the S-Labs program. Many students have utilized the program for their theses or dissertations, while others have acted as project managers even after graduation.

Gloyd noted two other challenges for Chinese universities to expand their contributions to global health research and practice. First is the language barrier. Chinese is not as widely spoken globally as English and effective professional and personal communication is critical. Secondly, global health work requires practitioners to adapt to different living and working environments to integrate into recipient countries effectively. However, Chinese people are generally less inclined to take such risks, work, and live in unknown places. This not only curtails their ability to integrate into and understand the complex landscape of the communities in which they work, but also limits the joy and enrichment of the experiences.

Despite these challenges, we believe that as more open-minded, adventurous, and internationally knowledgeable researchers and professionals emerge from Chinese universities, they will increasingly continue to join in global health practice. With time, Chinese universities will have more outstanding global health practitioners who can contribute to advancing South-South cooperation and promoting equity and justice in global health.

## Conclusions

In this paper, we presented some strengths, initiatives, and challenges of China’s academic global health in addressing health inequalities in the context of decolonization trends, drawing on Gloyd’s- and others’-experiences in global health education, practice, and research. To better respond to the impact of global health decolonization, we suggest that Chinese universities build an equity-focused global health curriculum, address power imbalances and inequalities in university-affiliated organizations, and strengthen South-South cooperation in practice. As China becomes increasingly involved in global health, we believe that a new generation of Chinese scholars, students, individuals, and organizations will emerge as carriers of unity and global health solidarity in the new era, working towards promoting global health fairness and justice.

## Data Availability

Not applicable.

## Abbreviations

HAI: Health Alliance International
SIGHT: Southern Medical University Institute for Global Health
CCUGH: Chinese Consortium of Universities for Global Health
GHRP: Global Health Research and Policy
UW: University of Washington
HICs: High-income countries
LMICs: Low- and middle-income countries
Africa CDC: Africa Centers for Disease Control and Prevention
AAC&U: Association of American Colleges and Universities
CUGH: Consortium of Universities for Global Health
NGOs: Non-governmental Organizations
GHSP: Global Health Support Programme
DFID: Department for International Development
SDC: Swiss Agency for Development and Cooperation
S-Labs: Silk Road Labs for Health Systems Strengthening
PIs: Principal Investigators
